# Apontic directly activates *hedgehog* and *cyclin E* for proper organ growth and patterning

**DOI:** 10.1038/s41598-017-12766-w

**Published:** 2017-09-29

**Authors:** Xian-Feng Wang, Yang Shen, Qian Cheng, Chong-Lei Fu, Zi-Zhang Zhou, Susumu Hirose, Qing-Xin Liu

**Affiliations:** 1 Laboratory of Developmental Genetics, Shandong Agricultural University, Tai’an, Shandong 271018 China; 2 Department of Developmental Genetics, National Institute of Genetics, Mishima, Shizuoka, 411-8540 Japan

## Abstract

Hedgehog (Hh) signaling pathway and Cyclin E are key players in cell proliferation and organ development. Hyperactivation of *hh* and *cyclin E* has been linked to several types of cancer. However, coordination of the expression of *hh* and *cyclin E* was not well understood. Here we show that an evolutionarily conserved transcription factor Apontic (Apt) directly activates *hh* and *cyclin E* through its binding site in the promoter regions of *hh* and *cyclin E*. This Apt-dependent proper expression of *hh* and *cyclin E* is required for cell proliferation and development of the *Drosophila* wing. Furthermore, Fibrinogen silencer-binding protein (FSBP), a mammalian homolog of Apt, also positively regulates *Sonic hh* (*Shh*), *Desert hh* (*Dhh*), *Cyclin E1* (*CCNE1*) and *Cyclin E*2 (*CCNE*2) in cultured human cells, suggesting evolutionary conservation of the mechanism. Apt-mediated expression of *hh* and *cyclin E* can direct proliferation of Hh-expressing cells and simultaneous growth, patterning and differentiation of Hh-recipient cells. The discovery of the simultaneous expression of Hh and principal cell-cycle regulator Cyclin E by Apt implicates insight into the mechanism by which deregulated *hh* and *cyclin E* promotes tumor formation.

## Introduction

Animal development requires the organ growth and patterning. How these two processes are coordinated remains poorly understood. The *Drosophila* wing is an excellent model to study the regulation of gene expression during the organ growth and patterning. The wing disc is a sac-like structure composed of disc proper (DP) cells and peripodial epithelium (PE). During larval development, both DP and PE cells proliferate extensively and are patterned, finally give rise to the adult wing^1–3^. The Hh signaling and Cyclin E can contribute to growth and patterning of the wing disc during development^4,5^.

Hh pathway is one of the major conserved signaling pathways that control animal development from *Drosophila* to humans, which has been implicated in stem cell maintenance, cell migration, axon guidance and tissue regeneration^6–9^. Most vertebrate species have three *hh*: *Shh*, *Indian hedgehog* (*Ihh*) and *Dhh*, each with different expression patterns and functions. In the *Drosophila* wing disc, morphogen Hh expresses in posterior (P) compartment cells and spreads into approximately 12 cells-wide of anterior (A) cells along A/P boundary, where it regulates target gene expression in the A compartment to control entire wing patterning through stabilizing full-length Cubitus interruptus (Ci^F^)^4,10–12^. Therefore, the expression of *hh* is vital during wing development. Engrailed (En) induces the expression of *hh* in the P compartment, at the same time, represses the Hh downstream component Ci^11,13^. Ci can exist in two forms: Ci^F^ and a repressor form (Ci^R^). Ci^R^ represses the expression of *hh* in anterior cells^12,14^. However, regulatory factor that directly activates *hh* transcription remained to be identified.

Cyclin E belongs to the cyclin family, which is required for cell division^15^. Dysregulation of *cyclin E* correlates with various tumors, including breast cancer and lung cancer^16,17^. Besides, deregulated Cyclin E activity causes cell lineage-specific abnormalities, such as impaired maturation due to unregulated cell proliferation^18^. In *Drosophila*, Cyclin E is essential for G1-to-S phase transition in the posterior cells of eye disc^19^. It has been reported that *cyclin E* is a potential target gene of Hh signaling in *Drosophila*. Hh pathway activates *cyclin E* expression through its unique transcription factor Ci in the posterior cells of eye disc^20^. In the wing disc, Hh pathway is turned on exclusively in the A cells near A/P boundary^21^. However, *cyclin E* expresses throughout the wing disc^5^. This contradiction suggests that other factors are involved in regulating the expression of *cyclin E*. Therefore, it is fruitful to investigate the regulation of *cyclin E* in the wing disc and the relationship between Cyclin E and Hh pathway.

Apt has been identified as a transcription factor involved in development of tracheae, head, heart and nervous system^22–25^. Apt can suppress metastasis^26^ and is required in the nervous system for normal sensitivity to ethanol sedation^27^. Moreover, Apt participates in JAK/STAT signaling pathway to limit border cells migration^28^. The human homolog of Apt, FSBP, is a cancer-related factor that is expressed in many tissues^29,30^. However, the role of Apt in the organ growth and patterning is unknown.

In this study, we unveiled a fundamental role of Apt in growth and patterning of the wing disc through coordinated expression of morphogen *hh* and cell cycle regulator *cyclin E*. Both loss of function and overexpression of *apt* resulted in defective wings. Further studies demonstrated that loss of *apt* function attenuated the expression of *hh* and *cyclin E*, while *apt* overexpression upregulated *hh* and *cyclin E*. Mutating the inherent Apt binding sites in the promoter region of *hh* and *cyclin E* compromised the expression of *hh* and *cyclin E*. Collectively, Apt directly activates the expression of *hh* and *cyclin E* to allow proper wing development. In addition, we found that Apt-dependent expression of *hh* and *cyclin E* is evolutionarily conserved in human cells.

## Results

### Apt is expressed in the wing disc and is required for wing development

**Figure 1:**
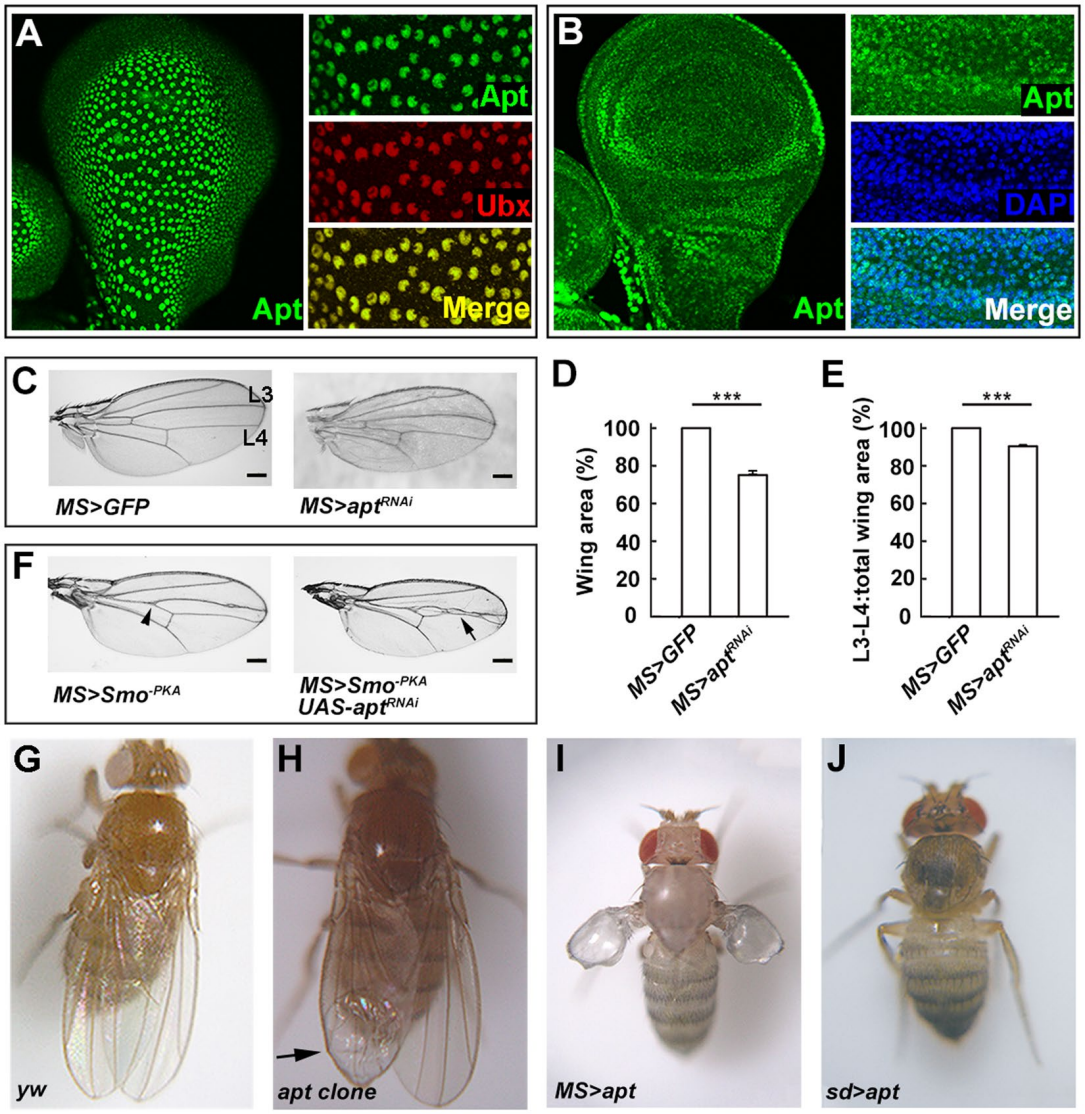
Apt is expressed in the wing disc and required for wing development. (**A** and **B**) A single optical section of the PE (**A**) or DP (**B**) of the wing disc from early third instar larva and the co-localization of Apt and Ubx (PE marker). (**C**) The adult wings of *MS* > *GFP* and *MS* > *apt* ^*RNAi*^. (**D**,**E**) Quantification of the wing size (**D**) and intervein region between L3 and L4 relative to total wing area (**E**) of *MS* > *GFP* and *MS* > *apt* ^*RNAi*^ by ImageJ. *MS* > *GFP* value was set as 100%. Error bars, SEM. Student’s t tests, ***p < 0.001. (**F**) Adult wings of the indicated genotypes. Apt knockdown enhanced the “fused wing” phenotype of *MS* > *Smo* ^*−PKA*^. Arrowhead indicates “fused wing” and arrow indicates enhancement phenotype of “fused wing”. (**G**–**J**) The adults of *yw* (**G**), *apt* ^*P*Δ*4*^ clones (**H**), *MS1096-GAL4; UAS-apt* (**I**) and *sd-GAL4; UAS-apt* (**J**). The arrow indicates the blistered wing. Scale bars, 200 um. Total numbers of analyzed wings were (**D**), 22; E, 31. Scale bars, 200 um.

As the first attempt to investigate the function of *apt* during wing development, we analyzed *apt* expression pattern in the wing disc by immunostaining using anti-Apt antibody. In the wing disc, Apt was detected in PE cells as revealed by co-localization with a PE marker Ubx (Fig. 1A). Apt was also detected in DP cells (Fig. 1B). These data clearly demonstrate that Apt is expressed in both the PE and DP of the wing disc, suggesting its possible role in wing development.

To analyze the role of Apt during wing development, we would examine the developing wing of homozygous *apt* null mutant. However, *apt* null homozygotes die as embryos^22^. Therefore, we firstly examined the phenotype of *apt* knockdown using an *MS1096*-*GAL4* driver. RNAi-mediated knockdown of *apt* resulted in a small wing, and also reduced the width between vein 3 and vein 4 (Fig. 1C–E). Overexpression of a dominant-negative form of Smoothened (Smo^−PKA^) caused a “fused wing” phenotype^31–33^. Knockdown of *apt* enhanced the “fused wing” phenotype (Fig. 1F, arrowhead indicates the “fused wing” phenotype and arrow indicates enhancement of the “fused wing” phenotype). Furthermore, we induced *apt* loss of function mutant clones in the wing disc using the *FLP/FRT* system^34^. The formation of these clones resulted in a small wing with a blistered phenotype (Fig. 1H) compared with the control wing (Fig. 1G). To investigate the effect of *apt* overexpression, we employed the *MS1096-Gal4* driver. Abnormal wings were induced by overexpression of *apt* (Fig. 1I). The wing was diminished and blistered, and the pattern of veins was disrupted and extra abnormal bristles were induced in the wing margin. In addition, when *apt* was overexpressed by a stronger driver (*sd-Gal4*), both wings and halters were lost (Fig. 1J). Taken together, the loss-of-function and overexpression analyses indicate that Apt is indispensable for wing development.

### Apt activates the expression of *hh* in the wing disc

**Figure 2:**
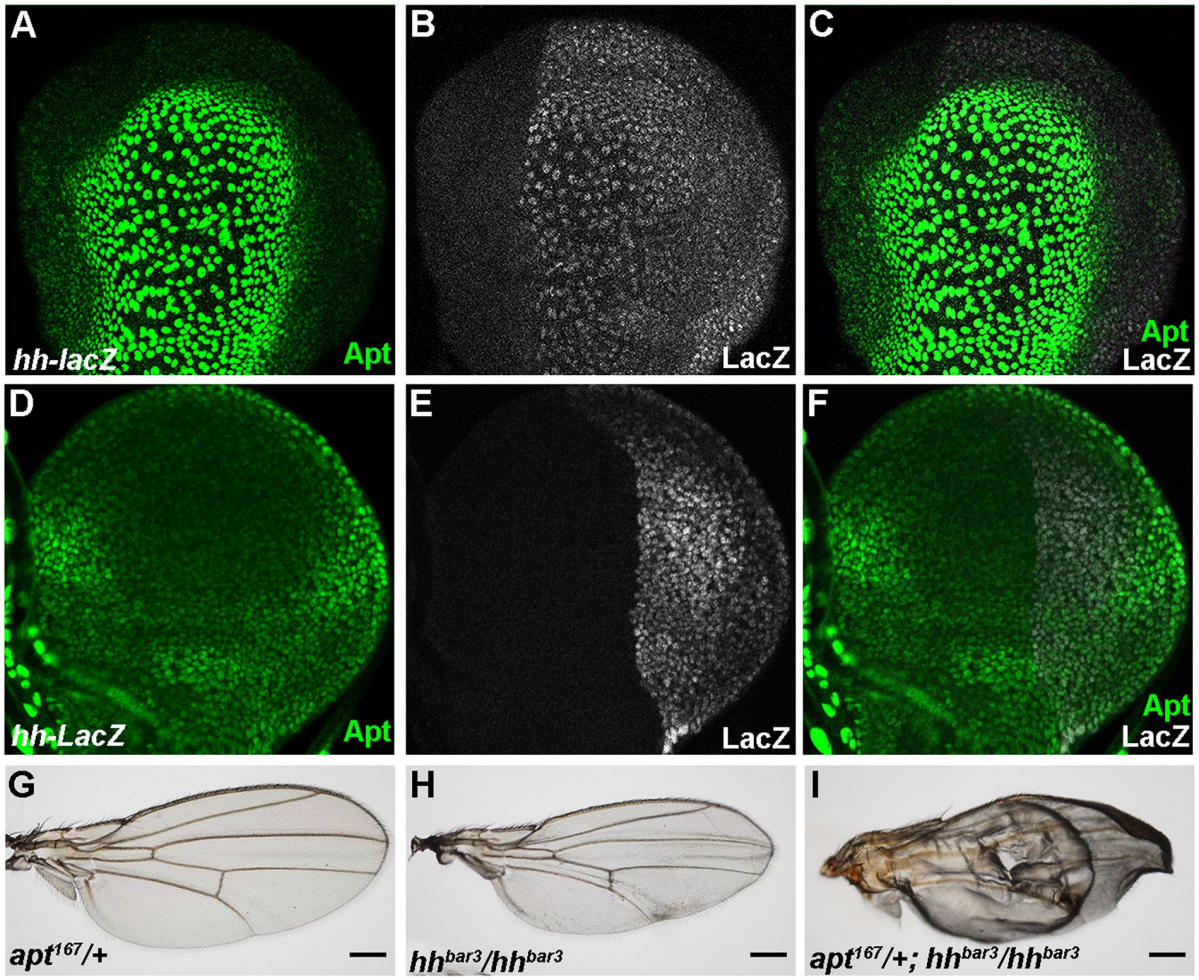
Expression and genetic interaction of *apt* and *hh*. (**A**–**F**) The expression of Apt and *hh-lacZ* in PE cells (**A**–**C**) and DP cells (**D**–**F**). (**G**–**I**) Genetic interaction between *apt* and *hh*. Adult wings of indicated genotypes. (**I**) Fifty percent of *apt* ^*PΔ4*^/+; *hh* ^*bar3*^/*hh* ^*bar3*^ wings exhibited patterning defects and blistered wing. Total numbers of analyzed wings were G, 37; H, 40; I, 113.

**Figure 3:**
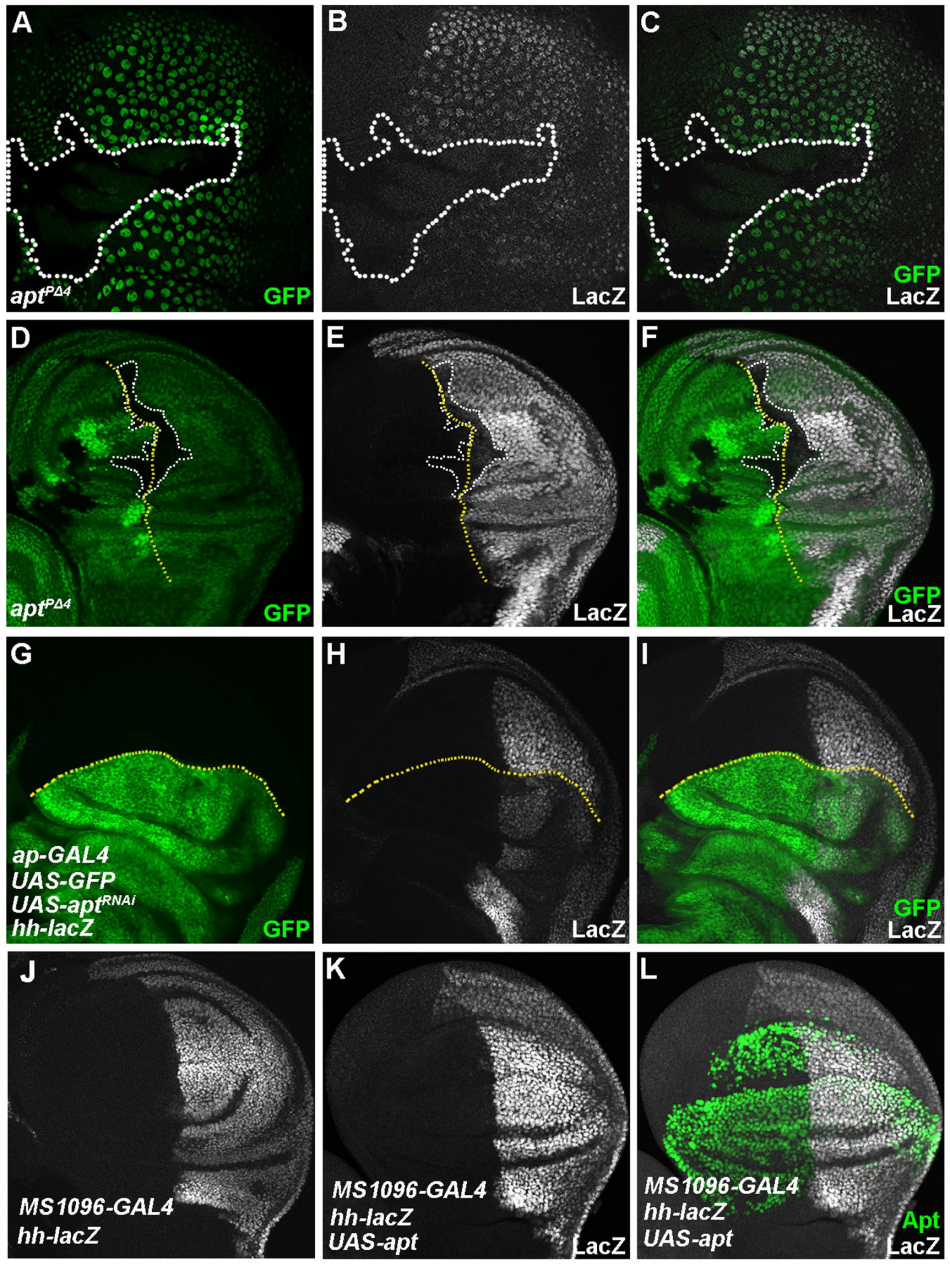
Apt regulates the expression of *hh*. (**A**–**F**) The decreased expression of *hh-lacZ* in the *apt* ^*PΔ4*^ clones of the PE (**A**–**C**) and DP (**D**–**F**). Clones are marked by white-dotted lines. The A/P boundaries are marked by yellow-dotted lines in **D**–**F**. (**G**–**I**) Knockdown of Apt using *ap*-*GAL4* driver resulting in the decreased expression of *hh*-*lacZ* in the dorsal region. The D/V boundaries are marked by yellow-dotted lines. (**J**–**L**) Overexpressed Apt - increased the expression of *hh* (**K**).

Given that the space between vein 3 and vein 4 is a characteristic monitor of Hh activity^21,35^, the observed narrowing the space between vein 3 and vein 4 upon knockdown of *apt* (Fig. 1C–E) implies that Apt can modulate expression of *hh* in the wing disc. The enhanced dominant-negative phenotype of Smo^−PKA^ by knockdown of *apt* (Fig. 1F) supports the notion. To examine the relationship between *apt* and *hh*, we first compared the expression of *apt* and *hh*, and found that Apt and *hh-lacZ* were co-expressed in PE cells (Fig. 2A–C) and P compartment cells of the DP (Fig. 2D–F) in the early third instar larval disc. Furthermore, *apt* exhibited genetic interaction with *hh*. Ninety-seven percent of *hh* ^*bar3*^ mutant (n = 40) showed slightly reduced area between L3 and L4 (Fig. 2H) and the remaining three percent showed wing blistering phenotype (Supplementary Fig. S5B). While heterozygotes of *apt* null allele showed normal wings (Fig. 2G), the same heterozygotes under the *hh* ^*bar3*^ background exhibited more severe phenotypes of reduced L3-L4 area and smaller wing with blister (Fig. 2I), which reproduced the *apt* loss of function phenotype (Fig. 1H). Transheterozygotes of two sets of *hh* alleles (*hh* ^*bar3*^/*hh* ^2^ and *hh* ^*Mir*^/*hh* ^2^) showed a smaller wing with an extra crossvein (Supplementary Fig. S1A–E), demonstrating that it is a loss of function phenotype of *hh*. While wings of animal heterozygous for *hh* ^2^ or *apt* null mutant were normal, trans-heterozygotes of the *apt* null allele and *hh* ^2^ showed the same wing phenotype (Supplementary Fig. S1F–I). These results suggest that Apt regulates the expression of *hh*. To address the issue directly, we analyzed the expression of *hh* under loss-of-function and overexpression of Apt. The expression of *hh-lacZ* was significantly reduced in the *apt* mutant clones in the PE (Fig. 3A–C) and the DP (Fig. 3D–F). In addition, the expression of *hh-lacZ* also decreased in the *apt*-knocked down region (Fig. 3G–I). By contrast, overexpression of Apt increased the expression of *hh-lacZ* (Fig. 3J–L). Moreover, Apt regulates the mRNA levels of *hh* and its target *dpp* (Supplementary Fig. S2). These results demonstrate that Apt activates the expression of *hh*.

### Apt directly controls *hh* in the wing disc

**Figure 4:**
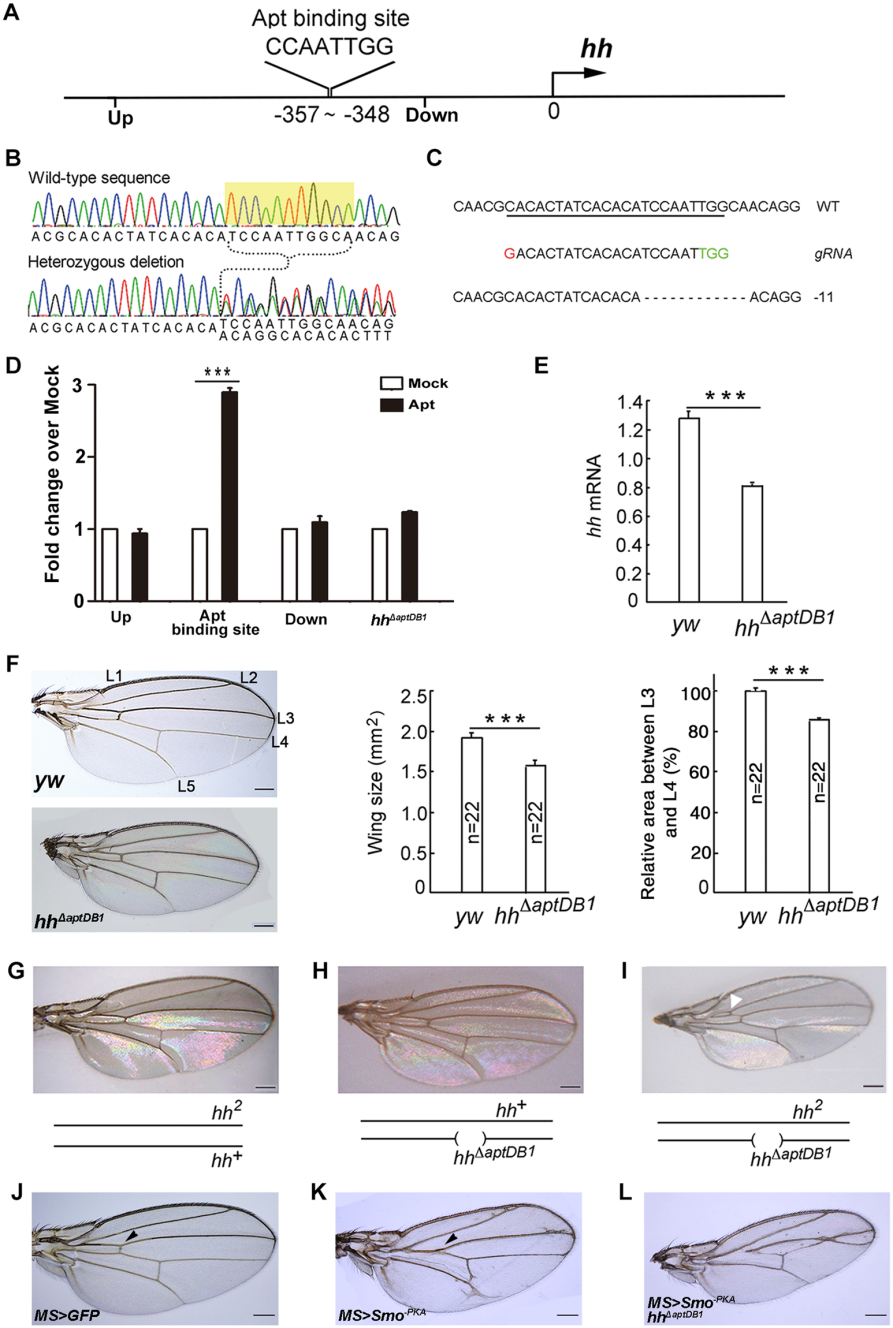
Apt directly regulates the expression of *hh* through its binding site in the *hh* promoter region. (**A**) Schematic representation of the Apt-binding site in the genomic sequence of *hh*. The arrow represents transcription start site and the numbers in base pairs are distance from the start site. Up and Down mean the region relative to the Apt binding site in the *hh* promoter region. (**B**) Sequences of a wild-type allele and a heterozygous mutant of *hh* ^*ΔaptDB1*^. The sequence of the mutant allele was inferred by subtracting a wild-type sequence from the mixed sequence. The deleted sequence is highlighted in yellow. (**C**) Cas9-induced mutagenesis at the *hh* locus. The *hh* locus in Cas9-induced mutants was PCR-amplified and sequenced. The wild-type sequence is shown at the top as a reference. The Cas9-gRNA target sequence is underlined with the protospacer-adjacent motifs (PAM) indicated in green. Deleted nucleotides in *hh* ^*ΔaptDB1*^ are shown as dashes. The deletion size is shown next to the sequence. (**D**) ChIP analyses of Apt at the *hh* promotor. Occupancies of Apt in binding site, upstream or downstream of wild type and binding site deletion mutant were analyzed by ChIP with Apt antibody. Data are normalized to Mock. (**E**) RT-qPCR analyses of *hh* mRNA in the wing disc of third instar larvae from *yw* or *hh* ^*ΔaptDB1*^. Error bars, SEM from three independent experiments. Student’s t tests, ***p < 0.001. (**F**–**L**) Deletion of the Apt-binding site in the *hh* promoter affects wing development. The wing size and the intervein region between L3 and L4 (control value was set as 100%) were decreased in *hh* ^*ΔaptDB1*^. Error bars, SEM. Student’s t tests, ***p < 0.001. *hh* ^2^/+ (**G**) or *hh* ^Δ*aptDB1*^/+ (**H**) adult wings show normal phenotype. All adult wings of *hh* ^*ΔaptDB1*^ */hh* ^2^ transheterozygotes exhibited abnormal morphologies in ACV (**I**). An arrowhead indicates the extra ACV. (**J**–**L**) The binding site deletion mutant enhanced the fused wing phenotype of *MS* > *Smo* ^*−PKA*^. Total numbers of analyzed wings were G, 157; H, 132; I, 74; J, 67; K, 80; L, 97. Scale bars, 200 um.

To address how Apt activates the expression of *hh*, we focused on a 15–kb region of the *hh* locus known to reproduce the normal *hh* expression pattern in the wing disc^36^. We identified one potential Apt binding sequence^25^ within the region (Fig. 4A). Chromatin immunoprecipitation (ChIP) assays using early third instar wing discs detected Apt protein on the predicted wild-type Apt binding site but not in the regions upstream and downstream of the binding site (Fig. 4A and D). We next assessed the function of the Apt-binding site in *hh* using a CRISPR-Cas9 system^37^. Since the designed gRNA contained the Apt-binding site, four Apt-binding site deletion mutants and two insertion mutants were generated (Fig. 4B and C; Supplementary Fig. S3A). The *hh* ^*ΔaptDB1*^ mutation abolished the occupancy of Apt on its binding site (Fig. 4D). Homozygotes of these mutations showed reduced expression of *hh* (Fig. 4E; Supplementary Fig. S3B) and exhibited the small wing and reduced vein 3–4 spacing phenotypes (Fig. 4F; Supplementary Fig. S3C and D). Effect of *hh* ^*ΔaptDB1*^ mutation on the *hh* function was also examined under the *hh* ^2^ heterozygous background. While wings of animals heterozygous for *hh* ^2^ or *hh* ^*ΔaptDB1*^ were normal, transheterozygotes of *hh* ^*ΔaptDB1*^ and *hh* ^2^ showed the same extra vein phenotype (Fig. 4G–I) as did transheterozygotes of *apt*-null allele and *hh* ^2^ (Supplementary Fig. S1H). Besides, we also examined the effect of *hh* ^*ΔaptDB1*^ mutation on the *hh* function under expression of a dominant-negative form of Smoothened (Smo^−PKA^). While *MS* > *Smo* ^*−PKA*^ alone showed reduction of the intervein space between vein 3 and vein 4 (Fig. 4J and K, arrowhead), *hh* ^*ΔaptDB 1*^exhibited more severe defects and enhanced the “fused wing” phenotype of *MS* > *Smo* ^*−PKA*^ (Fig. 4L). Taken together, these data demonstrate that Apt directly activates transcription of *hh* in the wing disc for proper wing development.

### Apt activates the *cyclin E* expression in the wing disc

**Figure 5:**
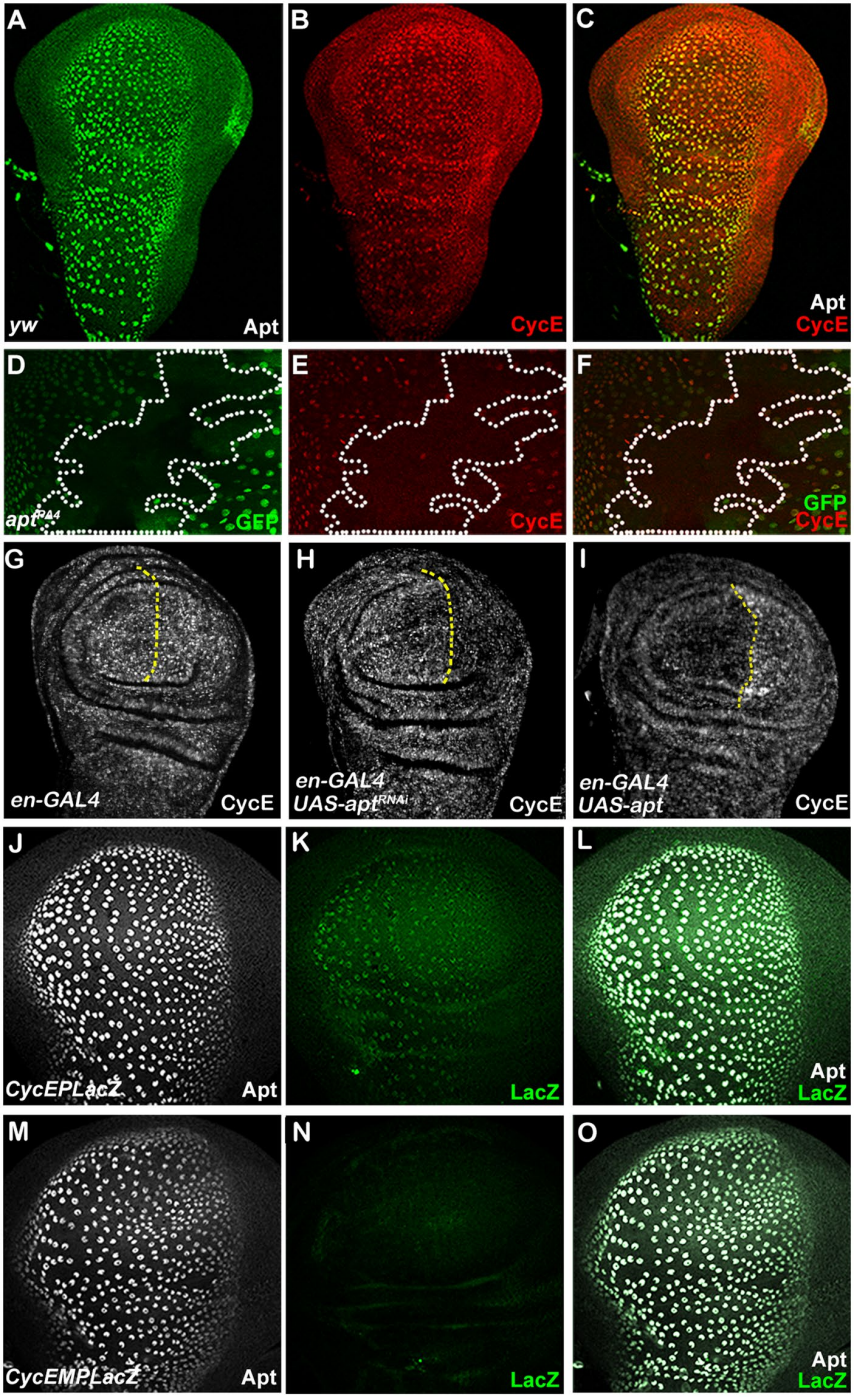
Apt controls the expression of *cyclin E*. (**A**–**C**) Apt and Cyclin E expression visualized by immunostaining with Apt antibody and Cyclin E antibody in a control wing disc. (**D**–**F**) Decreased Cyclin E expression (**E**) in the *apt* mutant clones (**D**). (**G**–**I**) Cyclin E expression in the wing discs of indicated genotypes. The A/P boundaries are marked by yellow-dotted lines. (**H**) Knockdown of Apt with *en*-*GAL4* decreased the expression of Cyclin E in the P compartment. (**I**) The expression of Cyclin E was increased in the P compartment from *en* > *apt* fly. (**J**–**L**) The reporter *cycEPlacZ* (**K**) was co-expressed with the endogenous Apt (**J**) in the wing disc. (**M**–**O**) Base substitutions in the Apt-binding site in *cycEMPlacZ* abolished the lacZ expression (**N**).

We have reported that Apt induces the *cyclin E* expression in the eye disc^38^. Therefore, we examined whether Apt consistently regulates *cyclin E* in the wing disc. To do this, we performed a double-staining experiment using Apt antibody and Cyclin E antibody. In the wild-type wing disc, Apt and Cyclin E were co-expressed (Fig. 5A–C). Furthermore, the expression of Cyclin E was significantly reduced in the *apt* mutant clones (Fig. 5D–F). Compared with control disc (Fig. 5G), *apt* knockdown decreased Cyclin E level (Fig. 5H), while *apt* overexpression increased Cyclin E (Fig. 5I). In addition, the *cyclin E* mRNA levels were decreased and increased upon RNAi-knockdown and overexpression of *apt* in the wing disc, respectively (Supplementary Fig. S4). These results indicate that Apt activates the expression of *cyclin E* in the wing disc.

### Apt directly controls *cyclin E* in the wing disc

Since Apt directly activates the expression of *cyclin E* in the eye disc, we anticipated a direct role of Apt in the expression of *cyclin E* also in the wing disc. This expectation was verified by transgenic reporter assays. The reporter gene^38^ carries the endogenous promoter and the *cyclin E* regulatory element containing a wild-type Apt-binding site (*cycEPlacZ*) or a mutated site (*cycEMPlacZ*). *cycEPlacZ* with the wild type binding site recapitulated the *cyclin E* expression in the wing disc (Fig. 5J–L). However, base substitutions in the Apt-binding site in *cycEMPlacZ* abolished the lacZ expression (Fig. 5M–O). These results indicate that Apt directly activates *cyclin E* through its binding site in the regulatory region of *cyclin E*.

### Apt is a growth sensor to control organ growth and patterning

**Figure 6:**
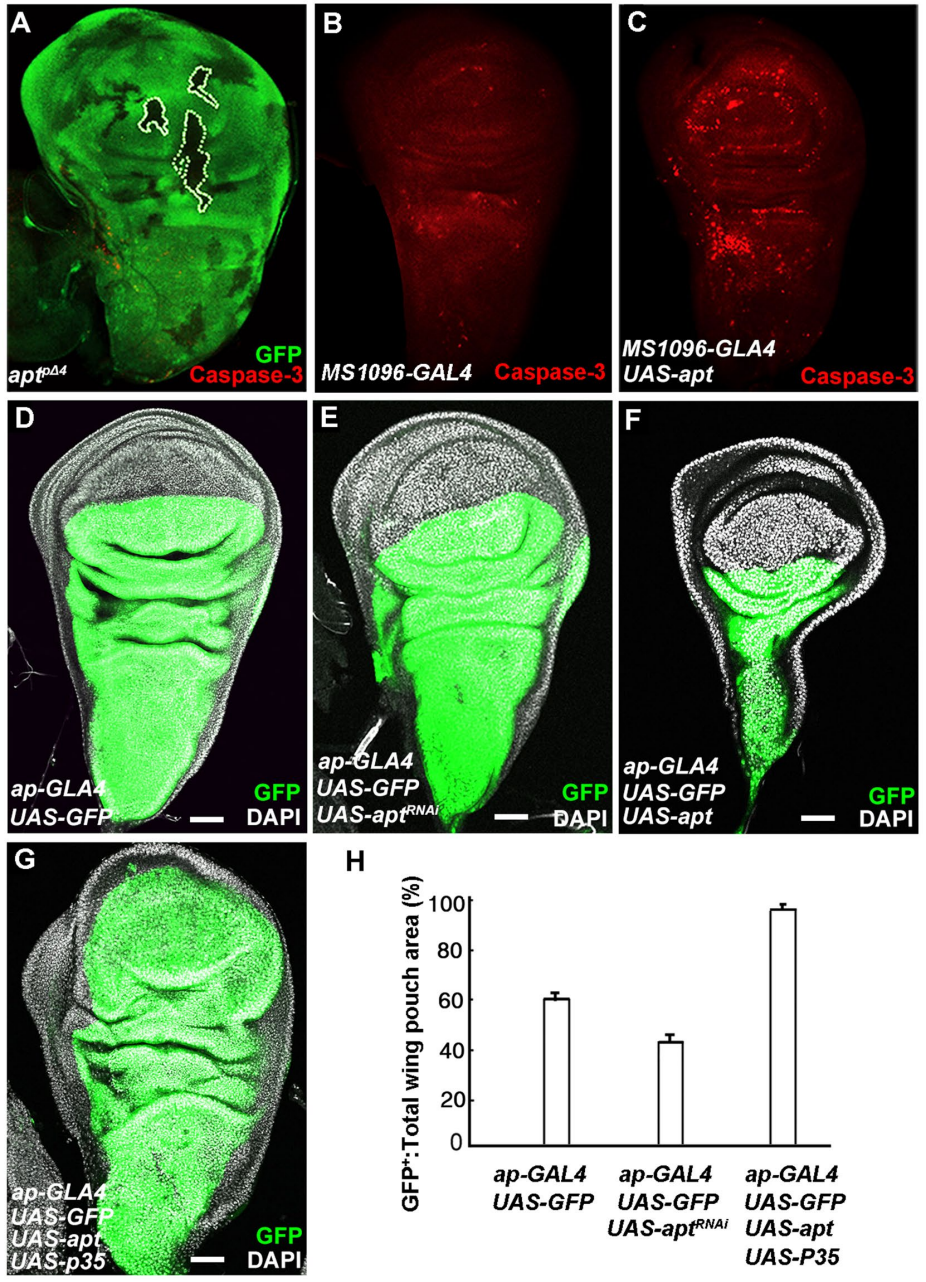
Apt as a growth sensor during development. (**A**–**C**) Wing discs from larvae of indicated genotypes labelled to visualize apoptotic cells with Caspase-3 antibody. Apoptosis was barely detectable in the *apt* mutant clones and their wild-type background (**A**) and in the disc from *MS1096*-*GAL4* (**B**). (**C**) Overexpressed Apt in the wing disc increased the number of apoptotic cells. (**D**–**H**) wing discs of indicated genotypes labelled to visualize GFP (Green) and DAPI (White). (**D**) Control disc. (**E**) Dorsal region of disc was reduced upon knockdown of Apt with *ap*-*GAL4* driver. (**F**–**G**) Overexpression of Apt reduced the disc (**F**), and simultaneous overexpression of *apt* and Caspase inhibitor P35 resulted in overgrowth of the wing disc (**G**). Scale bars, 50 um. (**H**) The ratio of GFP-positive wing pouch area to total wing pouch area decreased in the *apt*-RNAi disc, while it increased in the *apt* and p35-overexpressed disc.

Because both Hh and Cyclin E are involved in cell death and cancer^39–41^, we asked whether the overexpression phenotypes are caused by apoptosis. To test this, we investigated apoptosis in wing discs by staining with anti-Caspase-3 antibody. In the third instar wing disc, *apt* mutant clones showed few apoptotic cells (Fig. 6A). However, in the wing disc from an Apt-overexpressed larva, the number of apoptotic cells significantly increased compared with a control disc (Fig. 6B and C). This presumably explains why wing size was reduced upon strong overexpression of Apt (Fig. 1K and L). Besides, we examined the growth of wing discs upon *apt* knockdown in the dorsal region using an *ap-GAL4* driver. Compared with a control disc (Fig. 6D), *apt* knockdown region exhibited growth disadvantage (Fig. 6E and H). Overexpression of Apt using the *ap-GAL4* driver resulted in severely reduced dorsal region (Fig. 6F). When we inhibited cell death by simultaneous overexpression of a Caspase inhibitor P35, we observed outgrowth of cell layers from the disc in the *apt* and *p35* overexpressed region (Fig. 6G and H).

Homozygotes of *hh* mutations for the Apt-binding site exhibited the small wing but not the blistered phenotype. However, *hh* and *cyclin E* double mutant recapitulates the smaller and blistered wing. While *CycE* ^2^/+ flies showed normal wings, three percent of *hh* ^*bar3*^/*hh* ^*bar3*^ and eighteen percent of *CycE* ^2^/+; *hh* ^*bar3*^/*hh* ^*bar3*^ flies showed the smaller and blistered phenotypes (Supplementary Fig. S5A–C). We also observed genetic interaction between *hh* and *cyclin E* in the extra crossvein phenotype. While *CycE* ^*JP*^/+ and *hh* ^2^/+ flies showed normal wings, fifty-four percent of *CycE* ^*JP*^/+; *hh* ^2^/+ flies (n = 102) exhibited wings with the extra crossvein (Supplementary Fig. S5D–F). Collectively, these data suggest that Apt controls wing development by inducing appropriate amounts of Hh and Cyclin E.

### FSBP positively regulates *Shh* and *cyclin E* in human cells

**Figure 7:**
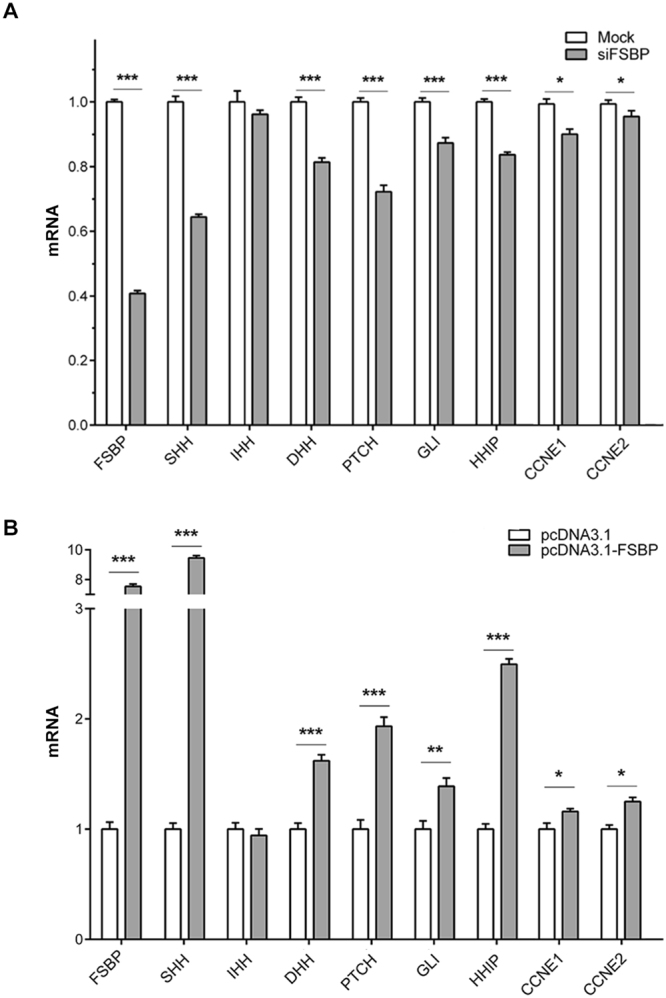
FSBP regulates *Shh* signaling and *cyclin E* in human cells. (**A** and **B**) 293 T cells were transfected with siFSBP or pcDNA3.1-FSBP and the levels of indicated mRNAs were quantitated. Data were normalized to GAPDH and shown as means ± SD from three independent experiments. P-values are Student’s t tests.

FSBP, the mammalian homologue of *Drosophila* Apt, is a cancer related factor. To examine whether FSBP regulates *Shh* and *cyclin E*, we used human 293T cells to knockdown or overexpress FSBP and analysed the mRNA levels of *Shh*, its signaling pathway genes and *cyclin E*. After transfection of FSBP siRNA, the mRNA level of FSBP decreased nearly 60 percent compared with mock. Under the condition, we observed marked decrease in the mRNA levels of *Shh* and *Shh* signaling pathway genes such as *Ptch*, *Gli* and *Hhip* (Fig. 7A). The levels of *cyclin E* (*CCNE1* and *CCNE2*) mRNA showed less prominent but statistically significant decrease. When we overexpressed FSBP, mRNA level of *FSBP* increased nearly 7.5 folds, and that of *Shh* increased dramatically 9 folds. The mRNA levels of *Shh* targets and *cyclin E* were also increased upon overexpression of FSBP (Fig. 7B). Interestingly, FSBP also regulates the expression of *Dhh*, but not *Ihh*. Taking together, these data suggest that the regulation of *hh*/*Shh* and *cyclin E* by Apt/FSBP is conserved from *Drosophila* to humans.

## Discussion

Morphogen Hh and cell cycle regulator Cyclin E control growth and patterning in vertebrate and invertebrate. Here, we unravel a fundamental role of transcription factor Apt/FSPB as a conserved regulator of *hh/Shh* and *cyclin E/CCNE*. During *Drosophila* wing development, Apt directly activates the expression of *hh* and *cyclin E* to control wing growth and patterning. Both loss-of-function and overexpression assays clearly demonstrated that Apt is vital for wing development. Further studies showed that loss of *apt* function attenuates, while overexpression of *apt* activates the expression of *hh* and *cyclin E*. Moreover, we found that the homolog of Apt, FSBP, can positively regulate *Shh* and its pathway genes, and *CCNE* in human cells.

Hyperactivation of Hh pathway and *cyclin E* has been implicated in many tumors^16,39^. In contrast, during development, cell proliferation must be precisely regulated and coordinated with the processes of cell patterning and differentiation, which are also regulated by Hh and Cyclin E^41,42^. This delicate balance is probably maintained by Apt-mediated proper expression of Hh and Cyclin E. Indeed, overexpression of Apt in the presence of apoptosis inhibitor P35 generated tumor-like outgrowth of cell layers in the wing disc. Apt-dependent expression of *hh* and *cyclin E* can direct proliferation of Hh-expressing cells and simultaneous growth, patterning and fate specification of Hh-recipient cells. Although mechanisms are quite different, this provides similar effects as an asymmetric division of a stem cell.

To assess the importance of the Apt-binding site in the promoter region of *hh*, we first tried a transgenic reporter assay. However, the regulatory region of *hh* encompassing the upstream region and the 1st intron (~15 kb)^36^ is too large to make a reporter construct for conventional P-element mediated transgenesis. Therefore, we employed the CRISPR-Cas9 system^37^ to mutagenize the endogenous Apt-binding site in the *hh* promoter. All 6 independent mutants exhibited the same phenotypes (reduced expression of *hh*, reduced wing size and the space between L3 and L4), suggesting that the observed phenotypes are not due to off-target effect of Cas9. Nevertheless, we inspected the possibility of off-target effect. Since our gRNA carries the binding sequence for Apt, a binding site of Apt in other than the *hh* promoter could be the most likely candidate for off-target. However, all the 6 mutants showed the wild type sequence around the Apt-binding site in the *cyclin E* promoter (Supplementary Fig. S6). Furthermore, we observed clear genetic interactions between *hh* ^*ΔaptDB1*^ and other *hh* mutants. Taken together, these data strongly suggest that the observed phenotypes are not due to off-target effect.

Although the expression of Apt and Hh overlapped in the P compartment of wing disc, how Apt specifically induces *hh* in the P compartment is still not clear. Since Ci^R^ has been known to repress the expression of *hh* in anterior cells^14^, Ci^R^ may interrupt the activation of *hh* by Apt in anterior cells. While our data strongly support that Apt is a transcription factor of *hh*, mutating the Apt-binding site on *hh* promoter alone induced the weak phenotype. However, the binding site mutation showed strongly enhanced “fused wing” phenotype in the background of overexpression of Smo domain-negative form (Smo^−PKA^). These observations suggest that besides Apt, other factor(s) might also regulate *hh* transcription during development. Therefore, both knockdown and overexpression of Apt only moderately affected the expression of *hh*. Hh, as an important morphogen, plays multifaceted roles in segmentation and wing patterning. Previous findings paid more attention on the protein modification of Hh but the regulatory mechanism underlying *hh* transcription was not well understood. Here we identified Apt as the first regulatory factor that directly activates *hh* transcription.

## Materials and Methods

### Fly stocks

All the adult phenotypes were obtained from females. Strains used were as follows. *apt* ^*PΔ4*22^, *apt* ^*p2*25^, *apt* ^167^ (gift from M. Starz-Gaiano), *cycEPlacZ* and *cycEMPlacZ* ^38^, *UAS-apt* (gift of D. Montell), *UAS-GFP* (gift of Y. Hiromi), *MS* > *Smo* ^*−PKA*31,32^. *hh* ^*Mir*^ was obtained from *Drosophila* Genetic Resource Center. *hh* ^2^, *hh* ^*Mrt*^, *hh* ^*bar3*^ *, CycE* ^2^ *, CycE* ^*JP*^ *, hh-LacZ*, *MS1096-GAL4*, *sd-GAL4* (8609) and *ap-Gal4* were obtained from Bloomington *Drosophila* Stock Center. *UAS-apt* ^*RNAi*^ lines were obtained from Tsinghua Fly Center and Vienna *Drosophila* Resource Center. *CAS-0001, TBX-000*2*, TBX-0004, TBX-0008, TBX-0010* were obtained from NIG-FLY Stock Center.

### Clonal analysis

Homozygous *apt* loss-of-function clones were generated by *hs-FLP/FRT* recombination^34^. *FRT42D* and *apt* ^*PΔ4*^ */CyO* were recombined to generate *FRT42D*, *apt* ^*PΔ4*^. Six pairs of *FRT42D*, *apt* ^*PΔ4*^ cross to *Gla/CyO* were allowed to lay eggs in G418-containing medium, and then test each line with *apt* ^*P2*^ */CyO*. *hs-FLP; FRT42D*, *Ubi-GFP/CyO* crossed with *FRT42D*, *apt* ^*PΔ4*^ */CyO* were performed at 25 °C. Heat shocks were performed 32-56 hours after egg-laying for 1.5 hours at 37 °C.

### Generation of CRISPR constructs

To induce mutations in the Apt-binding site in the *hh* promoter region, we used a Cas9–gRNA system. We designed gRNA in the *hh* promoter region carrying the binding sequence of Apt (Fig. 3A). The corresponding sequence was introduced into the pBFv-U6.2 vector and the gRNA transgenic flies were generated as described^37^. gRNA females were crossed to Cas9 males to obtain the founder animals. Male founders were crossed to female balancer. Offspring male flies were balanced and stocked. Genomic DNA was extracted from each offspring male and used for molecular characterization. PCR primers were designed to construct gRNA expression vectors and to amplify the promoter region of *cyclin E* (Supplementary Table S1).

### Chromatin immunoprecipitation (ChIP)

ChIP assays of wing discs were performed as previously described^43^. Briefly, 100 early third instar wing discs were dissected in PBS and fixed by 1% formaldehyde at room temperature for 20 minutes. Sonicated chromatin was immunoprecipitated using 10 μl anti-Apt antibody. Quantitative PCR using 4 μl of the purified DNA.

### RT-qPCRanalysis

Total RNAs were prepared from the dissected tissues using an RNAprep Pure Tissue kit TIANGEN #DP431). cDNAs were synthesized using a Prime Script^TM^ II1^st^ strand cDNA synthesis kit (TaKaRa #6210A). The real-time qPCR was conducted with Bio-Rad CFX96 real-time system using a SuperRealPreMix Plus (SYBR Green) Kit (TIANGEN #FP205) in a 20 ul reaction containing 2 pmol of relevant primers. The amount of mRNA was normalized to that of control tubulin mRNA. PCR primers used are shown in Supplementary Table (Supplementary Table S1).

### Antibodies and immunohistochemistry

Staining of larval tissues was performed as described previously^38,44^. Larvae were dissected in PBS, fixed with 4% formaldehyde for 40 minutes on ice and then permeabilized for 15 minutes at room temperature in PBS containing 0.5% NP-40. The following primary antibodies were used in overnight incubations at 4 °C in blocking solution: rabbit anti-Apt (1:1000), rabbit anti-β-galactosidase (1:2000, Cappel), rabbit Caspase3 (1:50, Cell Signaling Technology), mouse anti-β-galactosidase (1:500, Sigma), mouse anti-Ubx (1:10, Developmental Studies Hybridoma Bank (DSHB)), goat anti-Cyclin E (1:200, Santa Cruz). The secondary antibodies used were as follows: Alexa 488 donkey anti-rabbit IgG conjugate (1:500, Molecular Probes), Alexa 488 donkey anti-mouse IgG (1:500, Molecular Probes), Cy3-conjugated donkey anti-mouse IgG (1:500, Sigma), Cy3-conjugated goat anti-rabbit IgG (1:500, CWBIO), bovine anti-goat IgG-CFL 555 (1:500, Santa Cruz). Mounting used VECTASHIELD Mounting Medium with DAPI (Vector Labs). The caspase-3 staining was did as described previously^45^.

### Cell culture and transfection

293 T cells were cultured in DMEM (Gibco) containing 10% fetal bovine serum and 100 U/ml of penicillin/streptomycin. For RNA interference experiment, FSBP siRNA was designed by ourselves, the sequences were: siFSBP-F: 5′-GCCUGGUAAGAGACAGGAAdTdT-3′, siFSBP-R: 5′-UUCCUGUCUCUUACCAGGCdTdT-3′. After cells were cultured for 24 h in 12-well plate, the culture medium was changed to serum-free medium. Mock is no siRNA treatment. siRNA duplexes were transfected at a final concentration of 20 nM using lipofectamine 2000 (Invitrogen) according to the manufacturer’s instructions. Cells are harvested for real-time PCR after cultured for 48–96 h with serum containing medium. For overexpression experiment, transfection was carried out using PEI (polyethylenimine) transfection method. 293 T cells were transfected in 60 mm plates with 5 µg plasmid pcDNA3.1-FSBP, which was constructed by our lab, and pcDNA3.1-HisA/V5 plasmid was transfected as control. Forty-eight to ninety-six hours after transfection, cells are harvested for real-time PCR analyses with standard protocols. The primers were used as showing in the Supplementary Table S1.

### Microscopy and image treatment

Images were acquired in Olympus FV1200 confocal microscope and Olympus cellSens, treated with Adobe Photoshop CS6 image programs. Wing size and space between vein 3 and vein 4 were measured on each picture using the ImageJ computer program.

### Statistical analysis

Results are given as means SEM; each experiment included at least three independent samples and was repeated at least three times. Group comparisons were made by two-tailed unpaired Student’s t-tests. *P < 0.05; **P < 0.01, and ***P < 0.001.

## Electronic supplementary material

Supplementary Information
