# Supplementary material for: Apontic directly activates hedgehog and cyclin E for proper organ growth and patterning
Source: Sci Rep. 2017 Sep 29;7:12470. doi: 10.1038/s41598-017-12766-w (PMC5622130; doi:10.1038/s41598-017-12766-w)
Supplement: Supplementary file 1 — Supplementary Information [file 41598_2017_12766_MOESM1_ESM.pdf]

Supplementary Information

Apontic directly activates *hedgehog* and *cyclin E* for proper organ growth and patterning

Xian-Feng Wang[1], Yang Shen[1], Qian Cheng[1], Chong-Lei Fu[1], Zi-Zhang Zhou[1], Susumu Hirose[2,*] and Qing-Xin Liu[1,*]

[1]Laboratory of Developmental Genetics, Shandong Agricultural University, Tai'an, Shandong 271018, China

[2]Department of Developmental Genetics, National Institute of Genetics, Mishima, Shizuoka 411-8540, Japan

[*]Corresponding authors: liuqingxin@sdau.edu.cn (Q.X.L.), shirose@nig.ac.jp (S.H.)

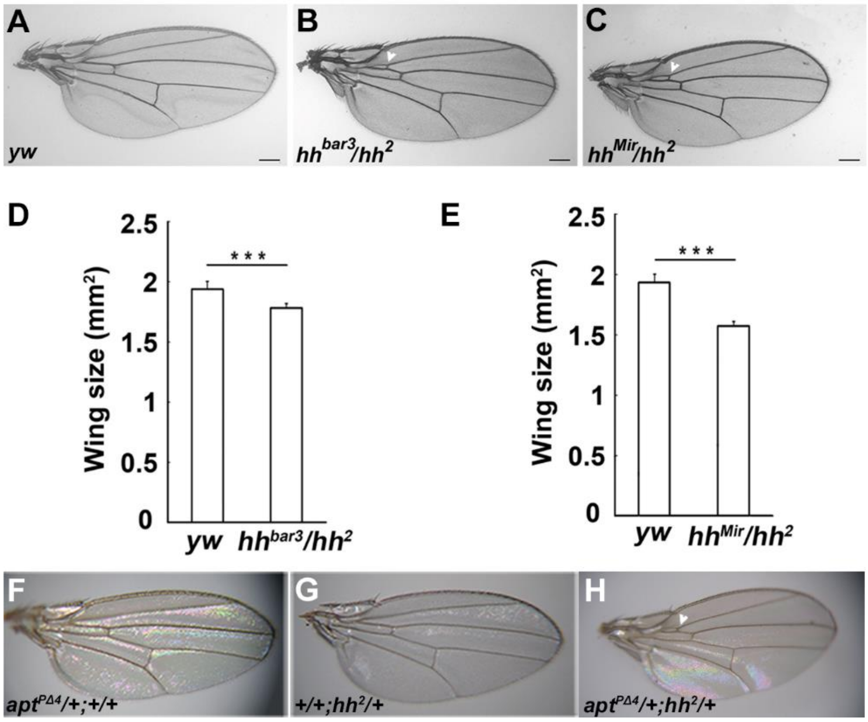

Figure S1. The wing phenotypes of *hh* mutants and genetic interaction between *apt* and *hh*. (**A**-**C**) Extra ACV in *hh* mutants. (**A**) Wild type (100%, n=102). (**B**) hh^{bar3}/hh^{2} (12.4%, n=105). (**C**) hh^{Mir}/hh^{2} (41.1%, n=56). Arrowheads indicate the extra ACV. (**D**, **E**) The wing size was decreased in the hh^{bar3}/hh^{2} (**D**) and hh^{Mir}/hh^{2} (**E**). (**F**-**H**) Genetic interaction between *apt* and *hh*. Adult wings of $apt^{P\Delta 4}$/+; +/+ (**F**) and +/+; hh^{2}/+ (**G**) showed normal pattern. (**H**) Forty percent of $apt^{P\Delta 4}$/+; hh^{2}/+ wings exhibited abnormal morphologies in anterior crossvein (ACV). An arrowhead indicates the extra ACV. Total numbers of analyzed wings were F, 158; G, 157; H, 116. Error bars, SEM. Student's t tests, ***$p < 0.001$.

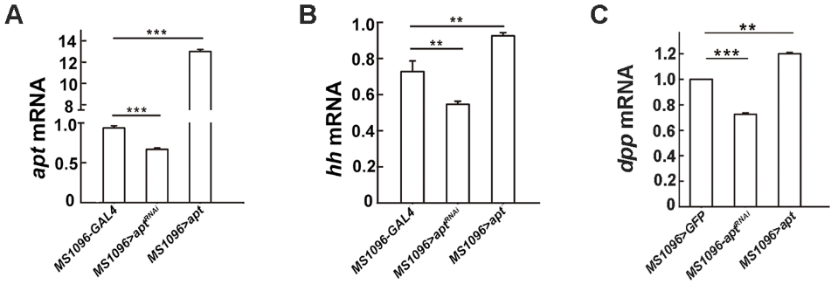

Figure S2. Apt regulates *hh* and its target *dpp*. RT-qPCR analyses of *apt* mRNA (**A**), *hh* mRNA (**B**) and *dpp* mRNA (**C**) levels in the wing disc from *MS1096-Gal4*, *MS1096-GAL4; UAS-apt*^{RNAi} or *MS1096-GAL4; UAS-apt*. Error bars, SEM from three independent experiments. Student's t tests, **p < 0.01, ***p < 0.001.

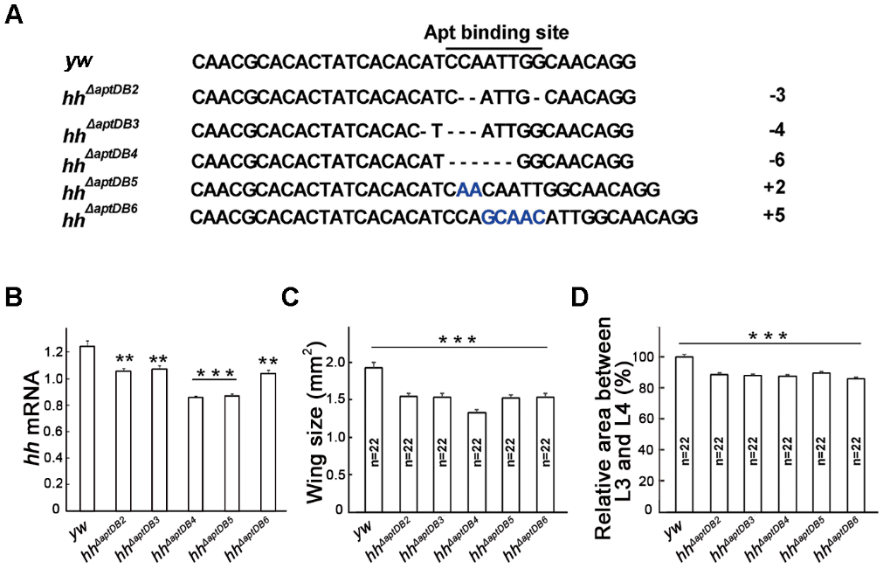

Figure S3. Apt-binding site mutations in the *hh* promoter affect *hh* expression and wing development. (**A**) Cas9-induced mutations at the *hh* locus. The wild-type sequence is shown at the top as a reference. Deleted nucleotides are shown as dashes. Inserted nucleotides are shown in blue. The deletion or insertion size are shown next to the sequence. (**B**) RT-qPCR analyses of *hh* mRNA level in the wing disc of third instar larvae from wild type or Apt-binding site mutants. Error bars, SEM from three independent experiments. Student's t tests, $^{**}p < 0.01$, $^{***}p < 0.001$. (**C**) The wing size was decreased in the Apt-binding site mutants. Error bars, SEM. Student's t tests, $^{***}p < 0.001$. (**D**) The intervein region between L3 and L4 (wild-type value was set as 100%) were decreased in the Apt-binding site mutants. Error bars, SEM. Student's t tests, $^{***}p < 0.001$.

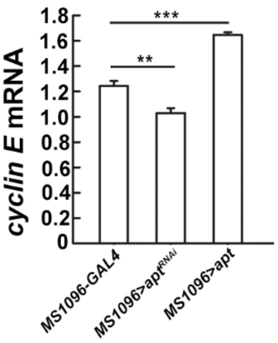

Figure S4. Apt controls the expression of *cyclin E*. RT-qPCR analyses of *cyclin E* mRNA level in the wing disc from *MS1096-Gal4*, *MS1096-GAL4; UAS-apt^{RNAi}* or *MS1096-GAL4; UAS-apt*. Error bars, SEM from three independent experiments. Student’s t tests, **$p < 0.01$, ***$p < 0.001$.

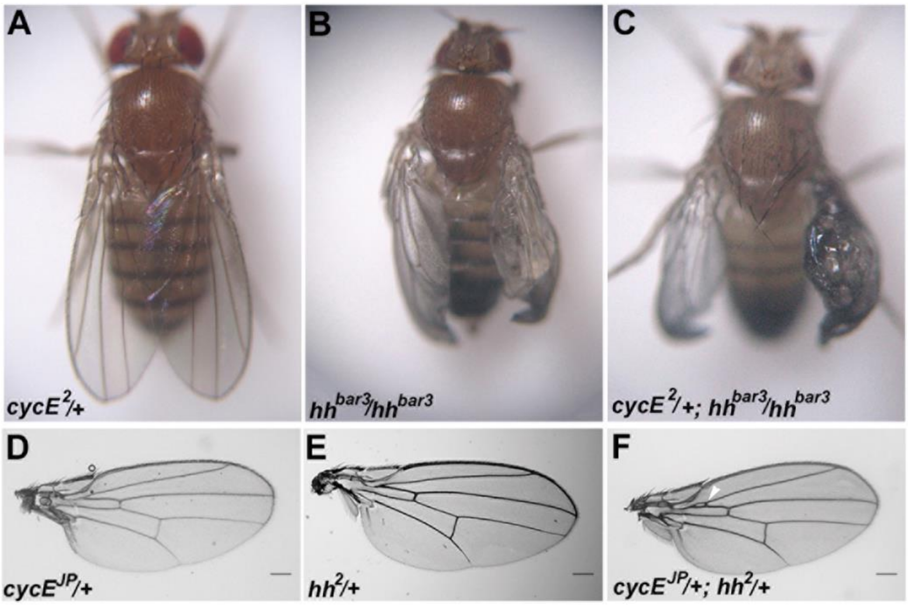

Figure S5. Genetic interaction between *hh* and *cyclin E*. (**A**-**C**) Adult wings of $cycE^{2}$/+; +/+ (**A**) showed normal pattern; Three percent of +/+; hh^{bar3}/ hh^{bar3} (**B**) and eighteen percent of $cycE^{2}$/+; hh^{bar3}/hh^{bar3} (**C**) showed the blistered phenotype. (**D**-**F**) Adult wings of $cycE^{JP}$/+; +/+ (**D**) and +/+; hh^{2}/+ (**E**) showed normal pattern. (**F**) Fifty-four percent of $cycE^{JP}$/+; hh^{2}/+ wings exhibited abnormal morphologies in the ACV. An arrowhead indicates the extra ACV. Total numbers of analyzed wings were A, 58; B, 40; C, 22; D, 108; E, 110; F, 102.

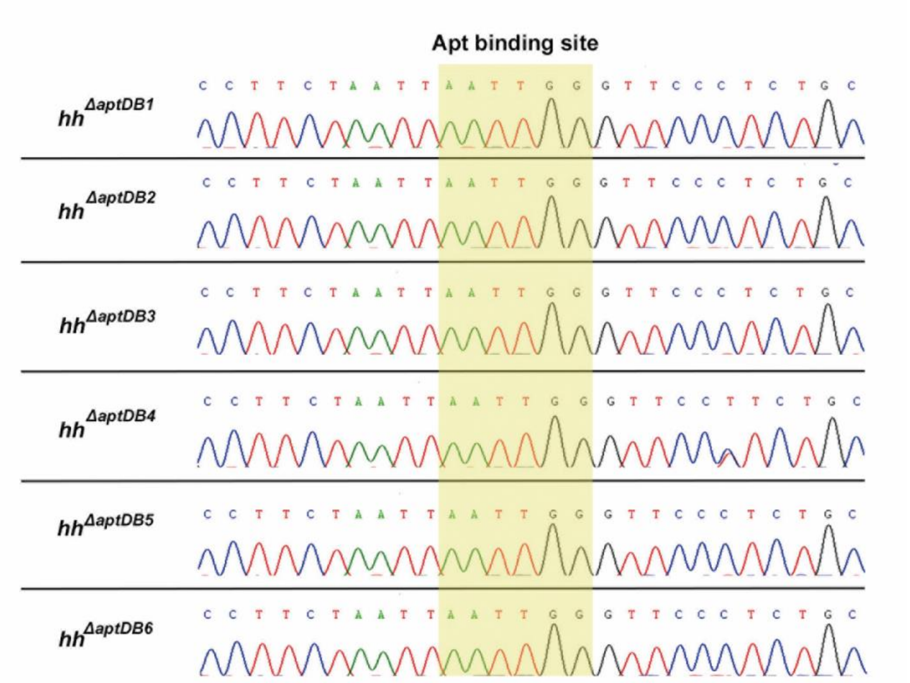

Figure S6. Sequence of the *cyclin E* promoter region carrying the Apt-binding site. Sequencing chromatograms in the *cyclin E* promoter region of $hh^{\Delta aptDB}$ mutants. The Apt binding sites in the *cyclin E* promoter were highlighted in yellow.

Table S1. List of oligonucleotides used in this study.

Name	Forward	Reverse
gRNA	CTTCGACACTATCACACATCCAAT	AAACATTGGATGTGTGATAGTGTC
hh promoter	AACGGAAGCAGCATTGAAC	TATGCCACTCGACGTTCGA
cycE promoter	AGTCATTTGTTTGCCAAGAG	TACGCAACGGATTGTCTATT
RT-qPCR *apt*	CGTCTCAGTGTGTCGCCTAA	CGTGGCGGATATGTTGTTCA
RT-qPCR *hh*	TCGTGTTTTGAGCATGACCG	AGCACCTGGTTCTTCTCCTC
RT-qPCR *dpp*	GGCTTCTACTCCTCGCAGTG	TCGAGGCTCCTACCGATCTA
RT-qPCR *cycE*	GCCATTCTTCCGAGTGATCT	GGCCATAAGCACTTCGTCAT
RT-qPCR *hh-PI*[a]	GGATGCTGCAGCTCTTCT	CAGATGTTGGATGGCATTGG
RT-qPCR *hh-up*[b]	GCACTTTTTCCGTTCCTT	TGTATTGTGTGACTCCTG
RT-qPCR *hh-down*[c]	GCAGCATAAAATGAACACCA	CACAAACACACACGCACA
RT-qPCR *FSBP*	CCCCAGATTTCCAGTTTTTGC	GTACCAGCCTGTGCCTCCT
RT-qPCR *CCNE1*	GGAGTTCTCGGCTCGCTCC	CGTCCTGTCGATTTTGGCC
RT-qPCR *CCNE2*	TTGACGATGTGCAGTTTTGGG	TCTTGGCCTGGATTATCTGGG
RT-qPCR *Shh*	GCGGACAGGCTGATGACTC	GTCACCCGCAGTTTCACTC
RT-qPCR *Ihh*	CGGGCCACTGGTTCATCAC	GATAGCCAGCGAGTTCAGGC
RT-qPCR *Dhh*	CGTGCCCAACTACAACCCC	CACATGTTCATCACGGCAATG
RT-qPCR *Ptch*	CCAGAAAGTATATGCACTGGCA	GTGCTCGTACATTTGCTTGGG
RT-qPCR *Gli*	AGCGTGAGCCTGAATCTGTG	CAGCATGTACTGGGCTTTGAA
RT-qPCR *HHIP*	CCCTGCATAGTGGGGATGG	AGGCTTAGCAGTCCTCTTTCAT
β-tubulin	AGTTCACCGCTATGTTCA	CGCAAAACATTGATCGAG
GAPDH	CATGAGAAGTATGACAACAGCC	AGTCCTTCCACGATACCAAAGT

a, *hh-PI*, *hh* promoter including Apt binding site.

b, *hh-up*, *hh* promoter upstream of Apt binding site.

c, *hh-down*, *hh* promoter downstream of Apt binding site.
